# Recurrent pulmonary arteriovenous malformation in a patient with Sjögren syndrome: A case report

**DOI:** 10.1097/MD.0000000000030954

**Published:** 2022-10-14

**Authors:** Yoon Mi Shin, Yook Kim, Jiyoul Yang, Bumhee Yang, In Ah Choi, Ki Man Lee

**Affiliations:** a Division of Pulmonary and Critical Care Medicine, Department of Internal Medicine, Chungbuk National University Hospital, Chungbuk National University College of Medicine, Cheongju, Republic of Korea; b Department of Radiology, Chungbuk National University Hospital, Chungbuk National University College of Medicine, Cheongju, Republic of Korea; c Division of Pulmonary and Critical Care Medicine, Department of Internal Medicine, Asan Medical Center, Chungbuk National University College of Medicine, Cheongju, Republic of Korea; d Division of Rheumatology, Department of Internal Medicine, Chungbuk National University Hospital, Chungbuk National University College of Medicine, Cheongju, Republic of Korea.

**Keywords:** case report, pulmonary arteriovenous malformation, Sjögren syndrome

## Abstract

Pulmonary manifestations of Sjögren syndrome (SS) are variable and may involve the airway or lung parenchyma and increase the risk of vascular and malignant disease. However, to date, only one case of pulmonary arteriovenous malformation (AVM) has been reported in a patient with SS. Here, we report a rare case of recurrent pulmonary AVMs with aggravating multiple cysts in a patient with SS during a period of 14 years.

**Patient concerns:**

A 45-year-old woman was diagnosed with SS and pulmonary AVM in the right lung. Her AVMs were embolized successfully and she was followed up annually for 14 years. Eleven years after the initial treatment, her chest computed tomography showed new pulmonary AVMs in the left lung with aggravating multiple cysts.

**Diagnosis:**

We diagnosed her with SS according to the American–European consensus group criteria of 2010. Chest computed tomography and angiographic findings confirmed the recurrence of pulmonary AVMs.

**Interventions:**

The patient’s recurrent pulmonary AVMs were successfully treated by embolization.

**Outcomes:**

Although her multiple cystic lung lesions had been aggravating during 14 years, she received embolization for the pulmonary AVMs twice and developed no complication related to these procedures. Currently, the patient is 56 years old and still alive with good performance state.

**Lessons:**

To date, only one case of pulmonary AVM has been reported in a patient with SS. The patient died 2.5 years after the diagnosis without recurrence of AVM. Here, we present a rare case of recurrent pulmonary AVMs associated with aggravating multiple cysts in both lungs, which were observed during long-term follow-up, in a patient with SS.

## 1. Introduction

Connective tissue diseases, such as rheumatoid arthritis, systemic lupus erythematosus, Sjögren syndrome (SS), scleroderma, mixed connective tissue disease, dermatomyositis, and polymyositis, are chronic autoimmune inflammatory disorders that can present with multiorgan involvement especially the lungs.^[1–6]^ Interstitial lung disease (ILD) and pulmonary hypertension are the most common lung manifestations in connective tissue diseases;^[6]^ however, any part of the respiratory system can be affected, including airway, lung parenchyma, vasculature, pleura, and respiratory musculature.^[3,7]^

SS is a lymphoproliferative disease with autoimmune features characterized by mononuclear cell infiltration of exocrine glands, notably the lacrimal and salivary glands.^[3,8,9]^ It is named after the Swedish ophthalmologist Henrik Sjögren (1899–1986). As SS is a multisystem disorder that is heterogenous in its presentation, course, and outcome, there is still no single clinical, laboratory, pathological, or radiological feature that could serve as a “gold standard” for the diagnosis and/or classification of this syndrome. Moreover, there have been several changes in the diagnostic criteria of SS. From 2002 to 2011, the American–European consensus group criteria was applied for the diagnosis of SS.^[10]^ Currently, the American College of Rheumatology/European League Against Rheumatism classification criteria is used in the diagnosis of primary SS.^[9]^

Lung involvement in SS has been well described, and negatively impacts the quality of life and increases mortality in patients with primary SS.^[7,11]^ The reported prevalence of lung involvement varies widely (9–75%) depending on the methods of detection and patient selection.^[12]^ In SS, variable involvement of the respiratory system has been reported, including the airway (bronchiolitis, bronchiectasis), parenchyma (ILD: nonspecific interstitial pneumonia, usual interstitial pneumonia, lymphocytic interstitial pneumonia, cysts, organizing pneumonia), pulmonary hypertension, increased risk of thromboembolic disease, and increased risk of developing lung malignancy. In rare cases, pulmonary amyloidosis and sarcoidosis have been reported.^[3]^

Here, we present a rare case of recurrent pulmonary arteriovenous malformation (AVM) in a patient with SS.

## 2. Case presentation

### 2.1. First admission (March 2007)

**Figure 1.**
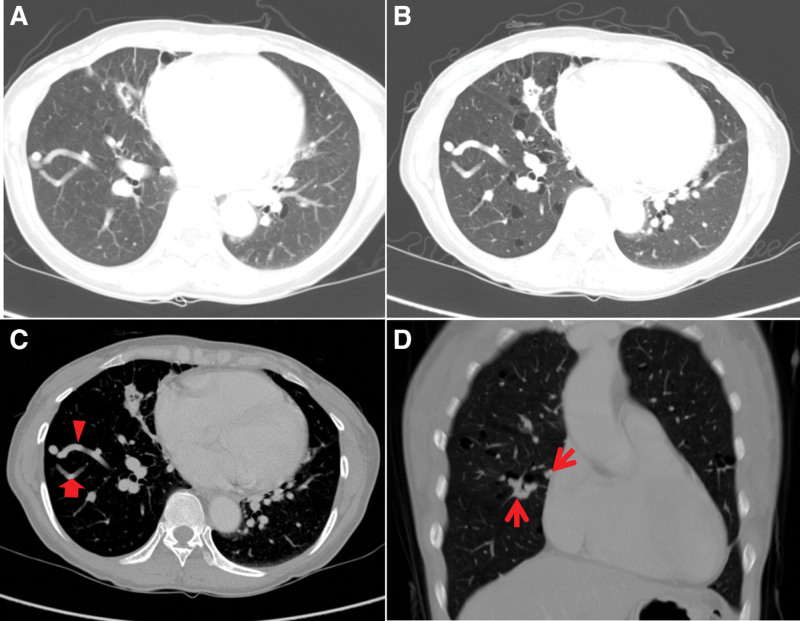
Initial chest CT image (A) and CT images after 3 years (B–D). (A) Initial chest CT image shows suspicion of AVM lesions in the RLL and small cystic lesions in both lungs. (B) Chest CT taken after 3 years reveals aggravating multiple cystic lesions and a persisting AVM lesion. (C) Chest axial CT image reveals multiple tubular lesions in the RLL and a feeding artery (red arrow) and draining vein (red arrowhead). (D) Another tubular enhancing lesion (red arrow) with the same finding as shown above are also identified in the right middle lobe on the coronal CT image. These findings are characteristic of pulmonary AVM. AVM = arteriovenous malformation, CT = computed tomography, RLL = right lower lobe.

A 42-year-old women complained of cough and sputum for 1 week prior to presentation to our center. She was referred from a primary clinic because of pneumonia. Her chest computed tomography (CT) image showed multiple cysts and pulmonary AVM in the right lower lobe (RLL) of the lung besides pneumonic consolidations (Fig. 1A). She recovered after 6 days and was discharged. We planned her follow-up in an outpatient clinic after 7 days, but the patient did not report for 3 years.

### 2.2. Second admission (November 2010)

After 3 years, the patient reported to our clinic and underwent another chest CT. Her chest CT images still showed pulmonary AVMs and aggravation of multiple cystic lung lesions. (Fig. 1B–D) She had mild oral and ocular sicca symptoms. We evaluated her laboratory markers of autoimmune disease. Her results of fluorescent antinuclear antibody and anti-ssA/Ro antibody test were positive, and we consulted a rheumatologist for the same. Her Schirmer’s test was positive and salivary scintigraphy showed abnormal findings. The diagnosis of SS was confirmed according to the American–European consensus group criteria, and she was administered pilocarpine.

**Figure 2.**
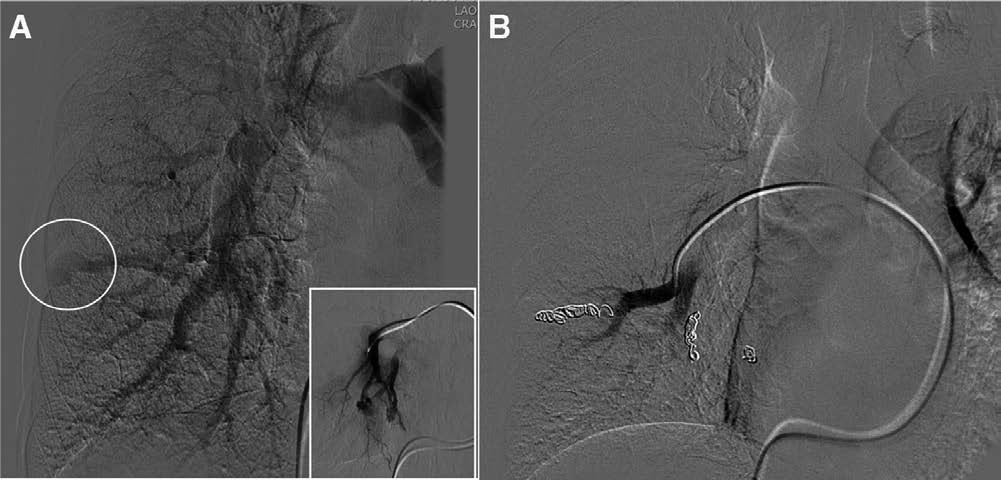
Initial angiographic findings. (A) Angiography of the right main pulmonary artery shows that the opacity was an (AVMs, white circle) in the right lower lobe. The selective angiogram of the right middle lobe segmental artery demonstrates another two AVMs (figure in white-border box on bottom left). (B) The final angiography recorded after coil embolization of the right middle and lower lobe AVMs confirms complete occlusion of the AVMs. AVMs = arteriovenous malformations.

We planned embolization of AVMs in the RLL and angiography was performed by an interventional radiologist (Fig. 2A), who found a total of three AVMs via the right pulmonary artery and embolized three feeding arteries using microcoils in the right middle lobe and RLL. (Fig. 2B) The patient was discharged without any complication and follow-up was planned with a rheumatologist.

### 2.3. Third admission (January 2012)

**Figure 3.**
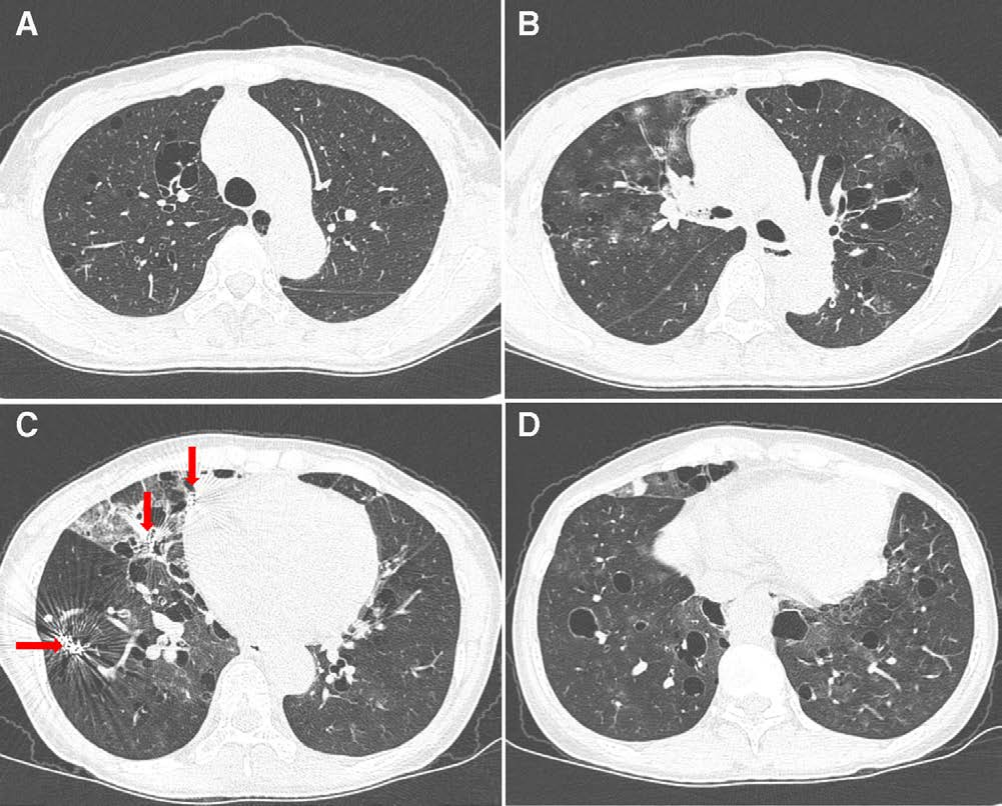
Chest CT performed after 5 years due to hemoptysis. Chest CT images showed multifocal ground glass appearances in both lungs and aggravating state of multiple irregular air-cysts with thin walls since 2010. Previous embolized coils are visible in the right middle lobe and right lower lobe (red arrows). CT = computed tomography.

The patient was readmitted due to hemoptysis. Her CT images showed multifocal ground glass appearances in both lungs and aggravating state of multiple irregular air-cysts with thin walls (Fig. 3A–D). Angiography was performed and the right bronchial artery was embolized using polyvinyl alcohol particles. The patient was discharged and was followed up in the pulmonology department with annual CT scans.

### 2.4. Last admission (November 2021)

**Figure 4.**
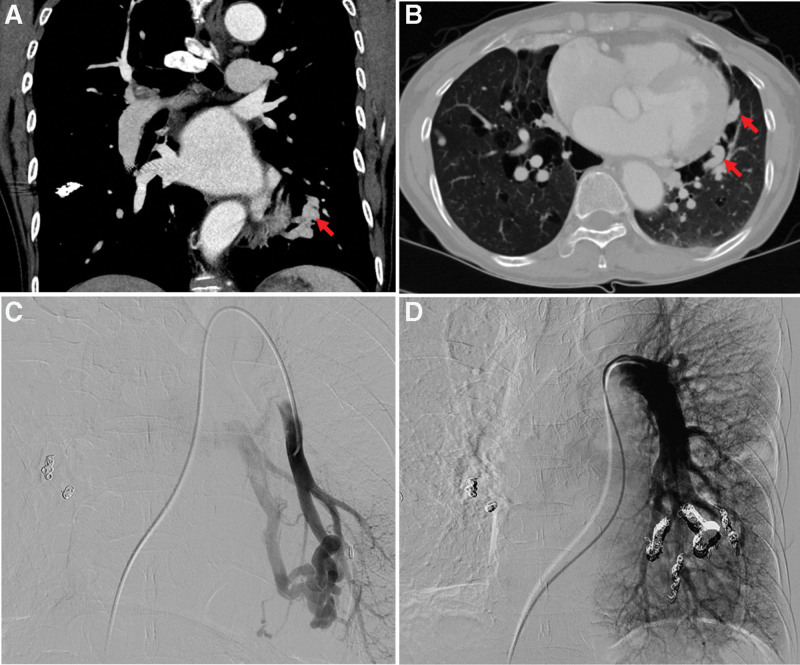
The follow-up chest CT images and angiographic findings 11 years after the first embolization. (A and B) Follow-up chest CT image 11 years after the first embolization demonstrates newly appeared multiple AVMs in the LLL of the lung, which were not seen in the initial chest CT scan. (C) The selective angiography of the left basal segmental pulmonary artery confirms the AVM in the LLL. After coiling of this branch, the posterior basal and inferior lingular AVMs were sequentially embolized with microcoils. (D) The final angiography demonstrates complete occlusion of the AVMs. AVMs = arteriovenous malformations, CT = computed tomography, LLL = left lower lobe.

Eleven years after the first embolization for AVM, the patient’s CT scan showed new AVMs in the left lower lobe. (Fig. 4A and B) In the angiography, four AVMs were identified in the left lower lobe. Embolization for the same was performed by the radiologist using various types of coils and gelfoam. (Fig. 4C and D)

## 3. Discussion

The estimated worldwide incidence of SS is seven cases per 100,000 population, with higher rates reported in Europe and Asia.^[8]^ The pulmonary manifestation of SS is variable. The airways are likely the most frequently involved area of the respiratory system in SS, including the nasal mucosa, trachea (xerotrachea), bronchi (xerobronchitis), or bronchioles. Therefore, cough is the most common complaint in patients with SS having a prevalence reported between 41% and 61%.^[3]^ The parenchymal involvement in patients with SS shows various types of ILD, including nonspecific interstitial pneumonia, usual interstitial pneumonia, lymphocytic interstitial pneumonia, organizing pneumonia, and cysts.

In SS, cysts tend to be thin-walled, variable in size, and have geographic implication of parenchymal architecture, producing a “dissolving lung appearance” with perivascular often basilar-predominant distribution, and frequent association with ground glass opacities and nodules.^[13]^

The vascular involvement of SS includes pulmonary hypertension and increased risk of thromboembolic disease. Patients with SS are at an increased risk of developing malignancies and the most common lung malignancy is non-Hodgkin’s lymphoma. Rarely, pulmonary amyloidosis and sarcoidosis have been reported.^[3,7]^

To date, only one case of pulmonary AVM has been reported in a Japanese woman with SS. She had multiple cystic lesions in both lungs and two pulmonary AVMs around the cystic lesions. The patient had received embolization, which was not successful and she developed rapidly progressive glomerulonephritis and died after 2.5 years. Her autopsy revealed that the lungs showed multiple cystic lesions of varying size and stenoses of the bronchiole lumen with hyperplasia of goblet cells, proliferation of smooth muscles, and retention of mucus in the airway lumen. The authors suggested that the stenosis of the bronchial lumens could have induced emphysema and destruction of the alveolar walls with a ball-valve mechanism. The histologic findings of pulmonary AVM showed an aggregate of veins and arteries around the bronchi and bronchioles. The etiology of the AVM could not be confirmed from histological findings.^[14]^ Gupta et al also described the eccentric vessel location of multiple cysts in patients with SS although they could not find any AVM.^[13]^ Our case is the second case of SS-associated pulmonary AVM reported globally and the first case of recurrence of pulmonary AVMs with aggravating multiple lung cysts in a patient with SS.

Our patient was diagnosed with SS according to the American–European consensus group criteria of 2010. She had ocular and oral symptoms, ocular sign (positive result of Schirmer’s test), oral sign (abnormal result of salivary scintigraphy), and autoantibody positivity (anti-ssA/Ro antibody test). These factors are also compatible with the diagnosis of SS according to the American College of Rheumatology/European League Against Rheumatism classification criteria.

We had followed up the patient for over 14 years and found aggravating multiple cystic lesions in both size and number and additional de novo pulmonary AVMs 11 years after the initial treatment. Her pulmonary AVMs were embolized successfully twice and she developed no complications related to these. We could not identify the exact etiology of pulmonary AVMs in our patient. However, according to a previous case series on multiple cysts in patients with SS and one case of AVM in a Japanese patient, we believe that the chronic inflammation of bronchioles could induce cystic changes in the lung parenchyma and location of adjacent vessels could lead to the occurrence of AVM in patients with SS.

Further case reports and studies are needed to identify the pathophysiology and clinical course of SS-associated pulmonary AVMs and cysts.

## Author contributions

**Conceptualization:** Yoon Mi Shin, Ki Man Lee.

**Data curation:** Yoon Mi Shin, Yook Kim, In Ah Choi.

**Formal analysis:** Jiyoul Yang, Bumhee Yang, In Ah Choi, Ki Man Lee.

**Investigation:** Yoon Mi Shin, Jiyoul Yang, Ki Man Lee.

**Writing – original draft:** Yoon Mi Shin, Yook Kim.

**Writing – review & editing:** Yoon Mi Shin, Yook Kim, In Ah Choi, Ki Man Lee.
